# The Effects of Cigarette Smoke Condensate and Nicotine on Periodontal Tissue in a Periodontitis Model Mouse

**DOI:** 10.1371/journal.pone.0155594

**Published:** 2016-05-20

**Authors:** Mikiko Kubota, Manabu Yanagita, Kenta Mori, Shiori Hasegawa, Motozo Yamashita, Satoru Yamada, Masahiro Kitamura, Shinya Murakami

**Affiliations:** Division of Oral Biology and Disease Control, Department of Periodontology, Osaka University, Suita, Osaka, Japan; Université de Lyon - Université Jean Monnet, FRANCE

## Abstract

Cigarette smoking is a major lifestyle-related risk factor for periodontal diseases. However, the pathophysiological role of cigarette smoking in periodontal disease has yet to be fully elucidated. Here we report that the systemic administration of cigarette smoke condensate or nicotine, which is the major ingredient of cigarette smoke, augmented alveolar bone loss. Concomitantly, the number of osteoclasts in periodontal tissues increased and the expression of receptor activator of nuclear factor κB ligand was upregulated at the ligated side in mice with periodontitis. Nicotine also attenuated alveolar bone repair after ligature removal. These observations highlight the destruction of periodontal tissue by smoking and the unfavorable clinical course of periodontal disease in patients with a cigarette smoking habit. The present study demonstrates that periodontal disease models are useful for elucidating the pathogenesis of cigarette smoking-related periodontal diseases.

## Introduction

Periodontal diseases are inflammatory disorders caused by the accumulation of a bacterial biofilm and characterized by the destruction of periodontal tissues [1, 2]. Although classified as bacterial infections, epidemiological studies have revealed that cigarette smoking is one of the major lifestyle-related risk factors for periodontal disease [3].

Cigarette smoking significantly increases the risk of developing various diseases including cancer, vascular disease, chronic obstructive pulmonary disease, as well as periodontal diseases [4–7]. It has been suggested that the increased incidence of these diseases in smokers may be due to chronic inhalation of chemicals in cigarette smoke that eventually alters the immune response [6]. Cigarette smoke contains more than 4000 chemicals, including 69 carcinogens [8]. When cigarette smoke is inhaled, the epithelial surface of the oral cavity, bronchi and lungs are exposed to high localized doses of nicotine [9]. Several *in vitro* studies have shown that nicotine can impair the migration, attachment and proliferation of gingival fibroblasts and periodontal ligament cells [10, 11] and alter the expression of neutrophil adhesion molecules [12]. Additionally, we previously reported that nicotine modulates the immunological characteristics of macrophages and dendritic cells [13, 14] and inhibits the differentiation of periodontal ligament cells [15].

Animal models are useful for investigating and understanding the pathogenic mechanisms of human diseases; however, smoking-associated periodontitis has not been investigated using mouse models. To investigate the effects of smoking on periodontal diseases in mouse models, we utilized nicotine, which has been well investigated and represents one of the main constituents of cigarette smoke, and cigarette smoke condensate (CSC), which is the total particulate component of cigarette smoke. Nicotine has been used in several animal studies; however, there are few studies that have compared the effects of nicotine with those of CSC on periodontal diseases [16]. Therefore, we examined the effects of nicotine and CSC on alveolar bone destruction in ligation-induced periodontitis and on bone repair after removal of the ligature in mice in this study.

## Materials and Methods

### Animals

Five-week-old C57BL/6 mice were purchased from Japan SLC Inc. (Shizuoka, Japan). Mice were maintained in the animal experiment laboratory of Osaka University Graduate School of Dentistry. All animal experiments were approved by the Institutional Animal Care and Use committee of Osaka University Graduate School of Dentistry (permit number: 24-012-0), prior to the commencement of experiments.

#### Experimental design 1: Ligation-induced periodontitis

**Fig 1:**
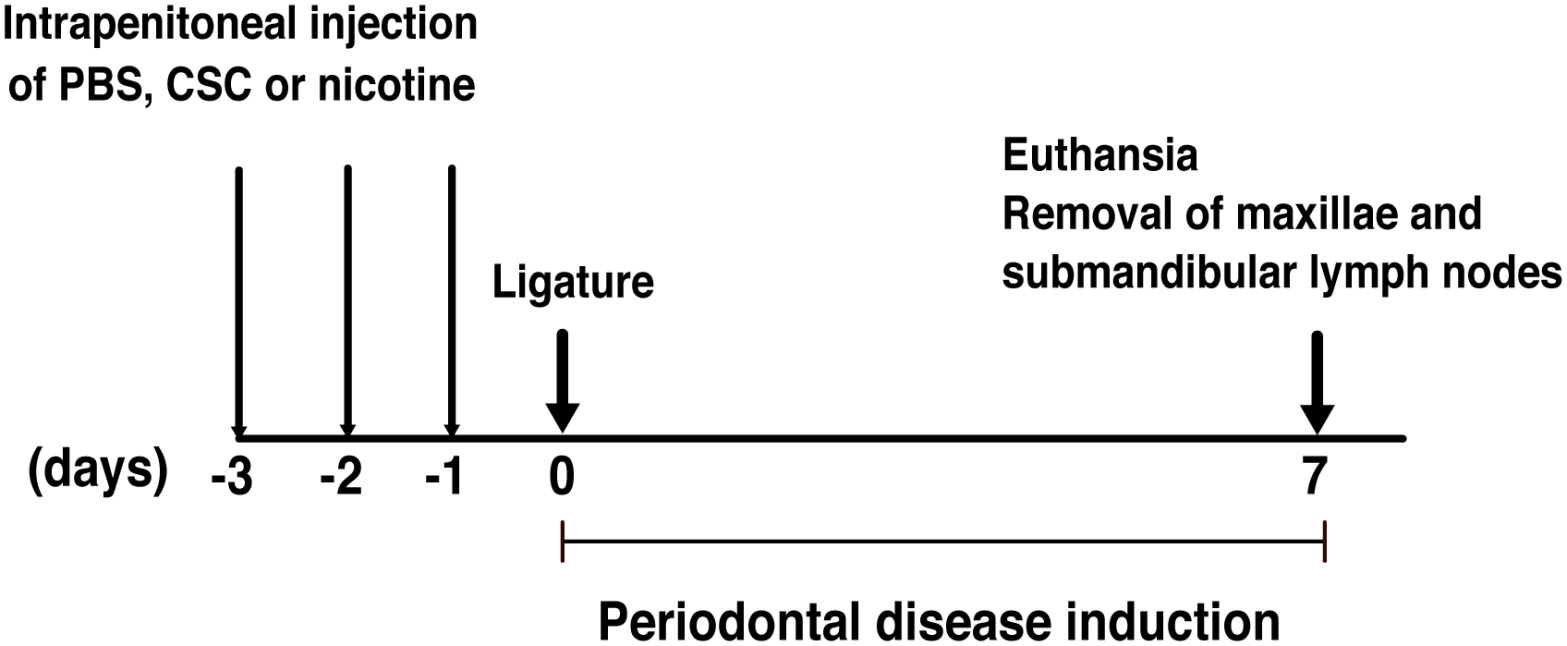
Overview of the experimental design for induction of periodontitis by ligature (Experimental design 1). Mice received daily intraperitoneal injections of PBS, CSC or nicotine for 3 consecutive days (days −3 to −1). Mice were examined on day 7 after placement of the ligature.

Twenty-one mice were divided into three groups. One group was injected with phosphate-buffered saline (PBS) as a control, and two groups were injected with either 720 μg of CSC per 20 g of mouse weight or 16 μg of nicotine per 20 g of mouse weight. CSC (Murty Pharmaceuticals, Lexington, KY, USA) was the total particulate matter prepared from a standard research cigarette (3R4F: University of Kentucky, KY, USA), and nicotine (Sigma, St. Louis, MO, USA) was diluted in PBS. The administered CSC and nicotine doses reflect the daily intake from 10 cigarettes [17]. Mice received daily intraperitoneal injections of PBS, CSC, or nicotine for 3 consecutive days (days −3, −2, and −1: Fig 1).

To induce periodontitis in mice, mice were anesthetized by intraperitoneal injection of sodium pentobarbital anesthesia (30 mg/kg), and all efforts were made to minimize suffering. A 5–0 silk ligature (Johnson & Johnson, New Brunswick, NJ, USA) was tied around the maxillary left second molar, in accordance with Abe et al [18], on day 0. The suture was gently tied to avoid damaging the periodontal tissue. The contralateral molar tooth in each mouse was not ligated as a control for measuring bone height. The ligations remained intact in all mice throughout the experimental period. After 7 days, mice were euthanized and the maxillae were removed for micro-computed tomography (CT) and histological analysis. In addition, submandibular lymph nodes were removed for real-time polymerase chain reaction (PCR) experiments. In our preliminary experiments, day 5 was not enough to induce disease compared with day 7, and some ligatures were spontaneously removed or loosened on days 8–10. Therefore, we determined that day 7 was optimal for inducing alveolar bone loss.

#### Experimental design 2: Periodontal bone recovery after ligature removal

Fifteen mice were divided into PBS-injected (n = 6) and nicotine-injected (n = 9) groups. Mice received daily intraperitoneal injections of PBS or nicotine for 3 consecutive days (days −3, −2, and −1), and then a 5–0 silk ligature was tied around the maxillary left second molar on day 0. Three of six PBS-injected and three of nine nicotine-injected mice were euthanized, and maxillae were removed for micro-CT analysis. The silk ligature was removed from three PBS-injected mice and three nicotine-injected mice on day 7. Mice were euthanized, and maxillae were removed for micro-CT analysis 10 days after ligature removal (day 17). The silk ligature of three nicotine-injected mice was removed, and these mice continued to receive nicotine injections on days 8, 9, and 10. Mice were euthanized, and the maxillae were removed for micro-CT analysis 10 days after ligature removal (day 17).

### Micro-computed tomography scanning for quantification of alveolar bone loss

**Fig 2:**
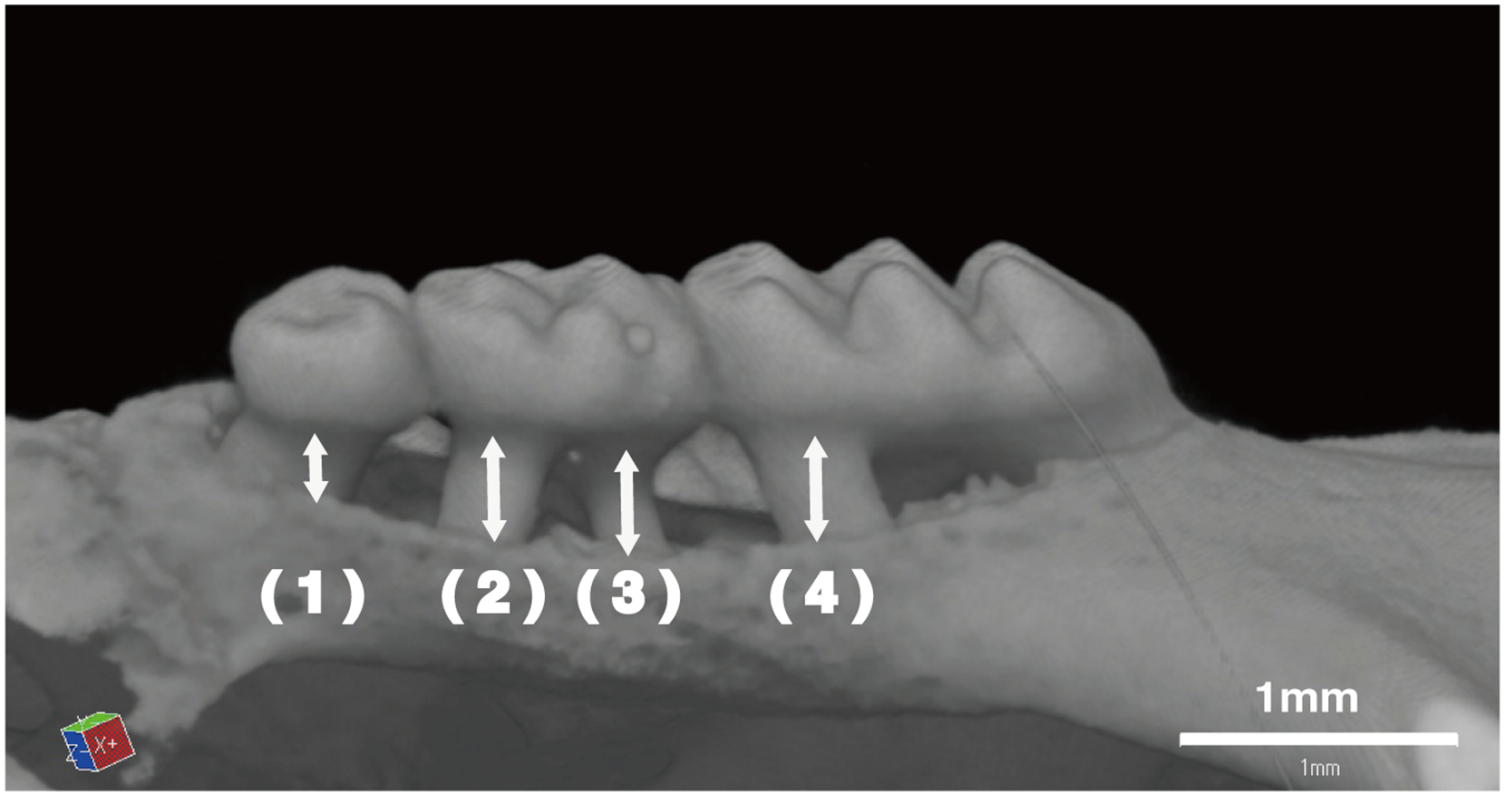
Schematic illustration of the measurement of the distance between the cemento-enamel junction (CEJ) and the alveolar bone crest (ABC). Distance from the CEJ to the ABC was measured at four points indicated on the first molar to the third molar.

To evaluate the alveolar bone loss, the alveolar bones including the maxillary second molar on the unligated (right) and ligated (left) sides were removed and observed using a R_mCT2 3D micro X-ray CT system designed for use with laboratory animals (Rigaku, Tokyo, Japan). Alveolar bone resorption was measured from CT images using the 3D image analysis software TRI/3D-BON (RATOC System Engineering Co., Ltd., Tokyo, Japan). Alveolar bone loss was measured from the cemento-enamel junction (CEJ) of the third mesial root to the alveolar bone crest (ABC) [shown in Fig 2 (1)] and from the distal and mesial root of the second and first molars [shown in Fig 2 (2), (3), and (4), respectively]. The alveolar bone loss of each group was defined as the sum of distances from the four sites [illustrated in Fig 2 (1) + (2) + (3) + (4)].

### Histological analysis

Maxillae from the mice were fixed in 4% paraformaldehyde (Wako Pure Chemical Industries Ltd., Osaka, Japan) overnight at 4°C and decalcified in decalcify solution B (Wako Pure Chemical Industries Ltd.) for 1 week. After decalcification, periodontal tissues were embedded in paraffin and sectioned at 5 μm in a mesio-distal orientation using a LEICA RA2245 microtome (Leica Microsystems, Wetzlar, Germany). Hematoxylin—eosin staining was performed using Mayer’s hematoxylin solution (Muto Pure Chemicals Co., Ltd., Tokyo, Japan) and 1% Eosin Y solution (Wako Pure Chemical Industries Ltd.).

### Tartrate-resistant acid phosphatase (TRAP) staining and osteoclast quantification

To detect TRAP-positive multinuclear cells, tissue sections were stained using the TRAP/ALP Stain Kit (Wako, Osaka, Japan) according to the manufacturer’s instructions. TRAP-positive cells on the alveolar bone surface surrounding the second molar were expressed as the mean of total numbers from five serial sections per mouse. Measurements were taken blind.

### RNA extraction and real-time polymerase chain reaction

Mononuclear cells from submandibular lymph nodes were prepared as previously reported with some modifications [19]. In brief, submandibular lymph nodes were removed and mononuclear cells were isolated by gentle mechanical dissociation through a 70-μm nylon mesh. Total RNA was isolated from mononuclear cells using a prepared phenol-chloroform solution (RNABee; Tel-Test Inc., Friendship, TX, USA) according to the manufacturer’s instructions. The precipitated RNA was dissolved in 0.1% diethylpyrocarbonate-treated distilled water. Complementary DNA synthesis and real-time PCR analysis were performed as previously described [20]. For real-time PCR, specific primers for mouse receptor activator of nuclear factor κB ligand (*Rankl*) and glyceraldehyde 3-phosphate dehydrogenase (*Gapdh*) were designed (Takara Bio Inc., Otsu, Japan) and the primer sequences were as follows: *Gapdh*, 5′-TGTGTCCGTCGTGGATCTGA-3′, 5′-TTGCTGTTGAAGTCGCAGGAG-3′ *Rankl*, 5′-AAACTGGTCGGGCAATTCTG-3′, 5′-AGGGTTGGACACCTGAATGCTA-3′. PCR reactions were carried out using the ABI 7300 Real-Time PCR System (Applied Biosystems, Foster City, CA, USA) using the SYBR green PCR master mix (Applied Biosystems) according to the manufacturer’s protocol. All reactions were run in triplicate.

### Statistical analysis

Data are summarized as the mean ± standard deviation (SD). Statistical analysis was performed using the Student *t*-test or analysis of variance and the *post hoc* Tukey test. *P* values < 0.05 were considered significant.

## Results

### Nicotine and CSC exacerbated silk ligature-induced alveolar bone loss

**Fig 3:**
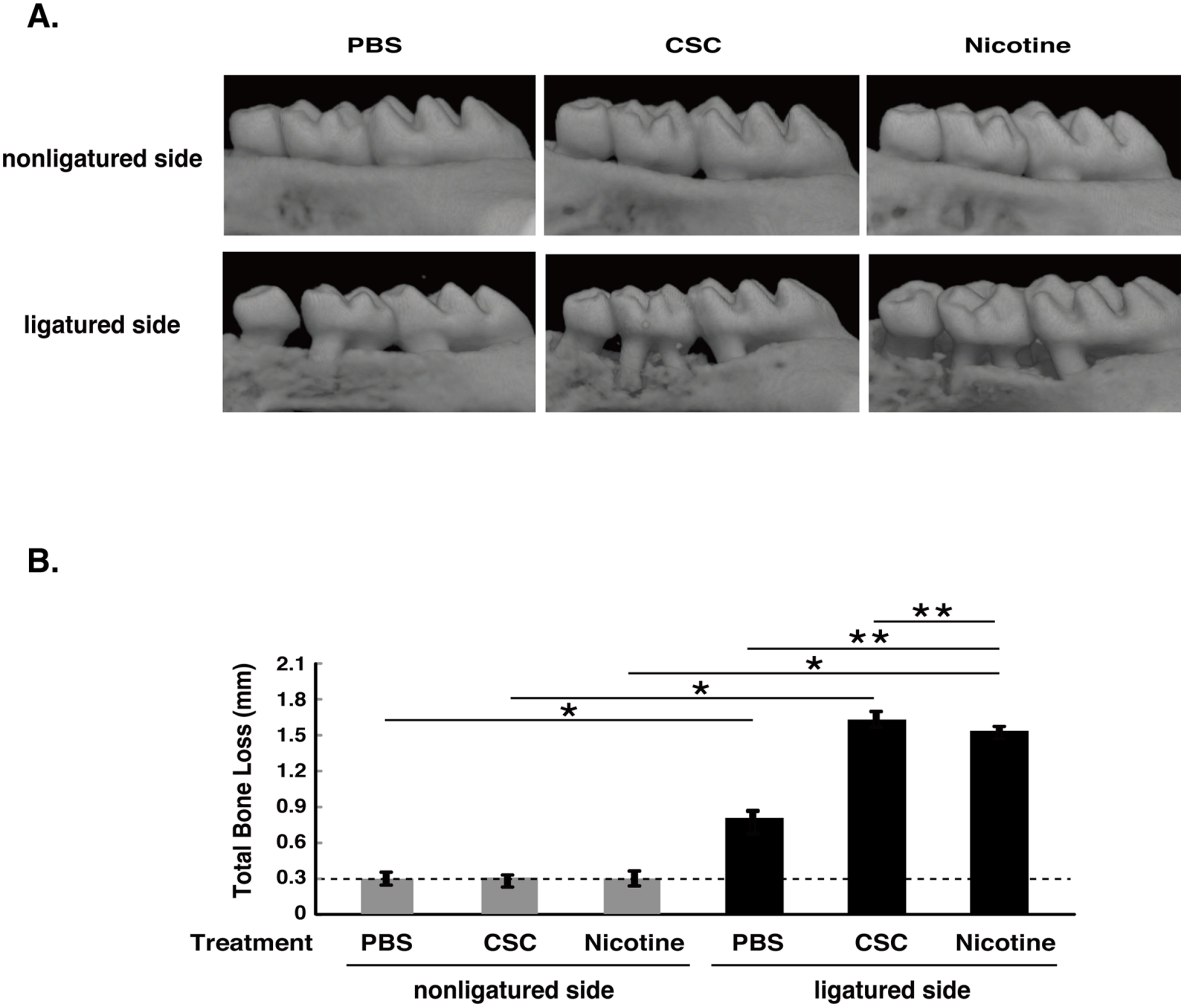
Micro-CT analysis. (A) The maxillae were scanned by micro-CT. (B) Measurement of total bone loss. Bar graphs represent the mean values ± SD of seven independent experiments. * *p* < 0.01, vs. without ligation. ** *p* < 0.01, vs. PBS-treated. Dashed line means baseline (unligated side in PBS treatment).

After 7 days of ligation, mouse maxillae were scanned by micro-CT to examine the alveolar bone. The total CEJ-ABC distances of the four sites were measured and calculated (Fig 3A and 3B). Ligation-induced alveolar bone loss was observed in PBS-treated (control) mice. Nicotine and CSC treatment markedly exacerbated ligation-induced alveolar bone loss compared with PBS treatment. We also confirmed that CSC (28.8, 144, and 720μg per 20 g of mouse weight) and nicotine (0.64, 3.2, and 16μg per 20 g of mouse weight) promoted alveolar bone destruction in a dose-dependent manner (data not shown).

### Nicotine and CSC treatment enhanced ligation-induced bone resorption

**Fig 4:**
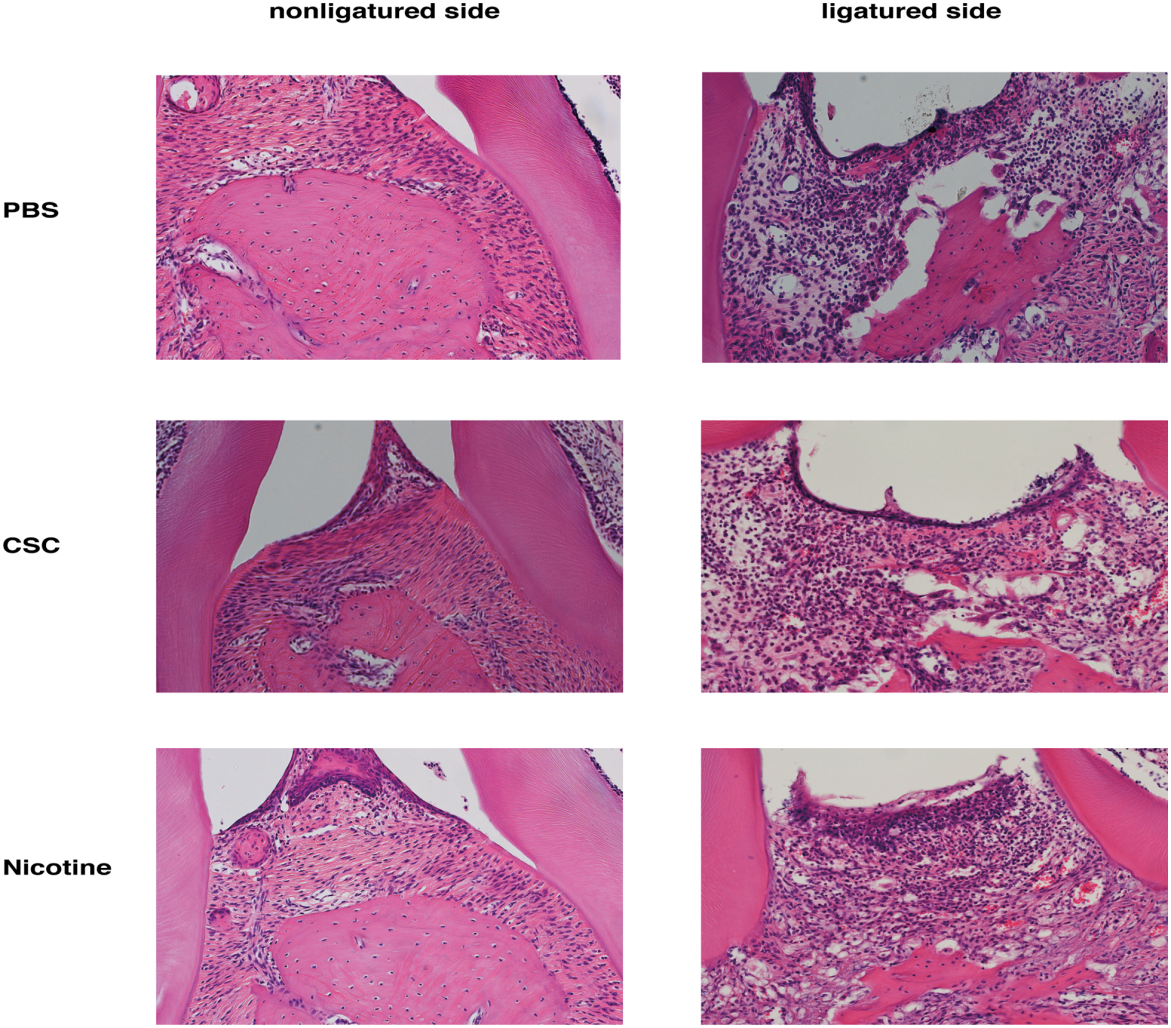
Histological analysis. Histological analysis of alveolar bone between the first molar and the second molar with or without a ligature. Decalcified periodontal tissue sections were prepared and stained with hematoxylin and eosin solution. Representative tissue sections are shown.

Alveolar bone surfaces were smooth regardless of nicotine or CSC treatment in mice without ligation (Fig 4), whereas alveolar bone surfaces were rough in mice with a ligature. Unexpectedly, although ligation led to a marked increase in inflammatory cell infiltration and alveolar bone destruction, the degree of inflammatory cell infiltration was not affected by PBS, nicotine, or CSC treatment.

### Nicotine and CSC accelerated ligature-induced osteoclastogenesis

**Fig 5:**
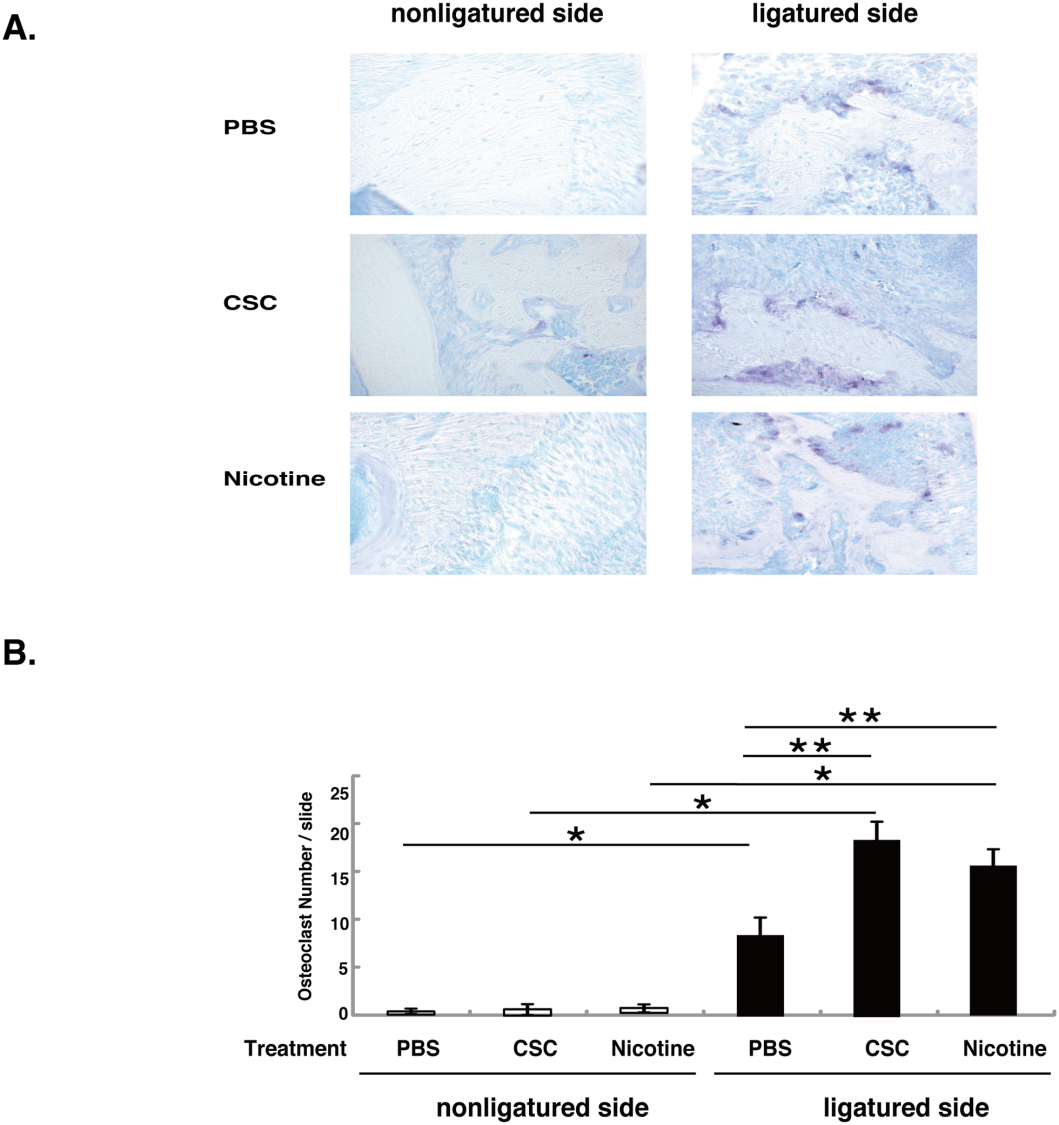
TRAP staining. (A) Detection of osteoclasts in tissue sections by tartrate-resistance acid phosphate (TRAP) staining. (B) Quantification of TRAP-positive osteoclasts. The mean of number of positive cells was determined from five serial sections per mouse. Data are represented as mean ± SD (n = 7). * *p* < 0.01, vs. without ligation. ** *p* < 0.01, vs. PBS-treated.

Few osteoclasts were observed near the alveolar bone surface in unligated tissues, whereas TRAP-positive cells were detected in the ligated tissue of PBS-treated mice (Fig 5A). The number of TRAP-positive cells in the ligated tissues was increased by nicotine or CSC treatment (Fig 5A and 5B).

### Ligature-induced RANKL expression was increased by nicotine and CSC

**Fig 6:**
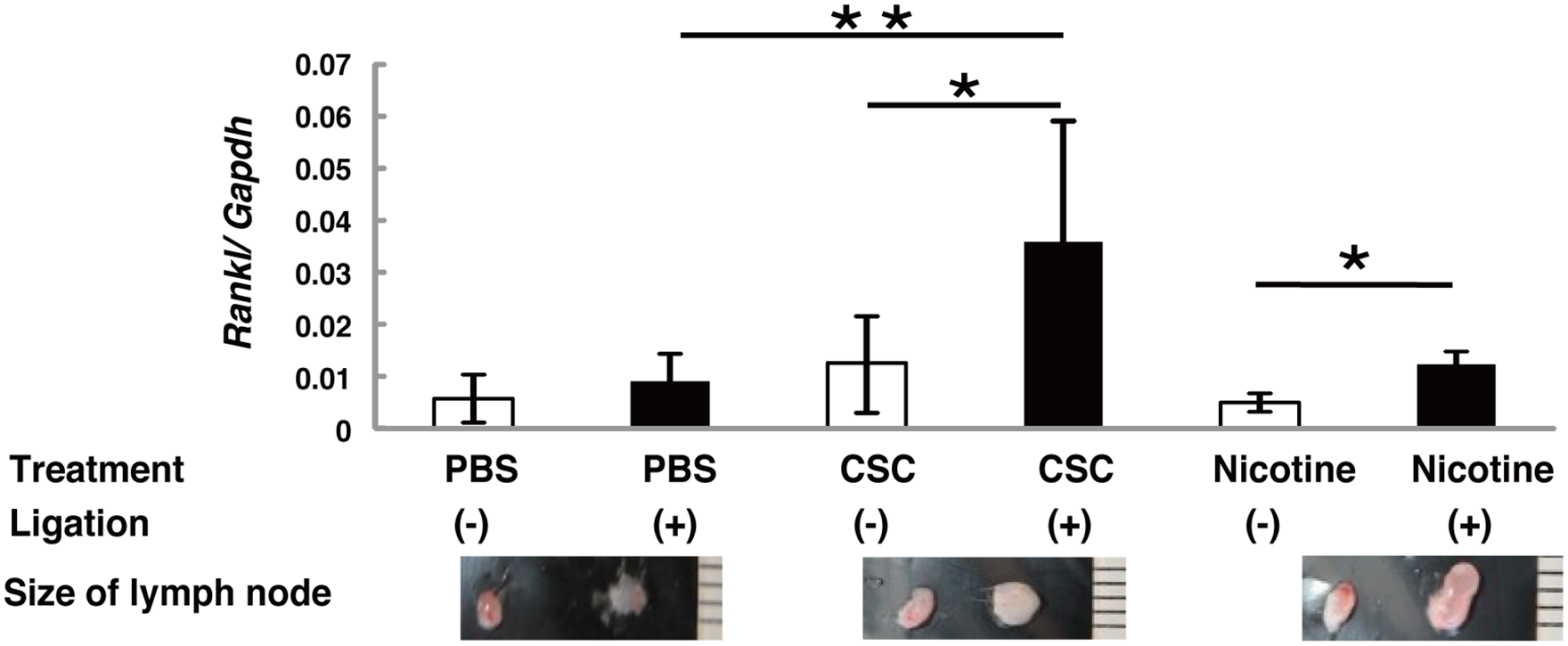
RANKL expression in submandibular lymph nodes. *Rankl* mRNA expression in submandibular lymph nodes was assessed by real-time PCR. Quantitative mRNA values were normalized to the amount of *Gapdh*. Bar graphs represent the mean values ± SD (n = 7). * *p* < 0.01, vs. without ligation. ** *p* < 0.01, vs. PBS treated. Lower panels are images of submandibular lymph nodes. Representative lymph nodes are shown.

*Rankl* expression was significantly elevated in the submandibular lymph nodes of ligated mice treated with nicotine or CSC, compared with the ligated PBS-treated mice (Fig 6). Furthermore, the submandibular lymph nodes in the ligated side were substantially larger in CSC and nicotine-treated mice (Fig 6: lower panels).

### Nicotine treatment suppressed alveolar bone recovery after ligature removal

**Fig 7:**
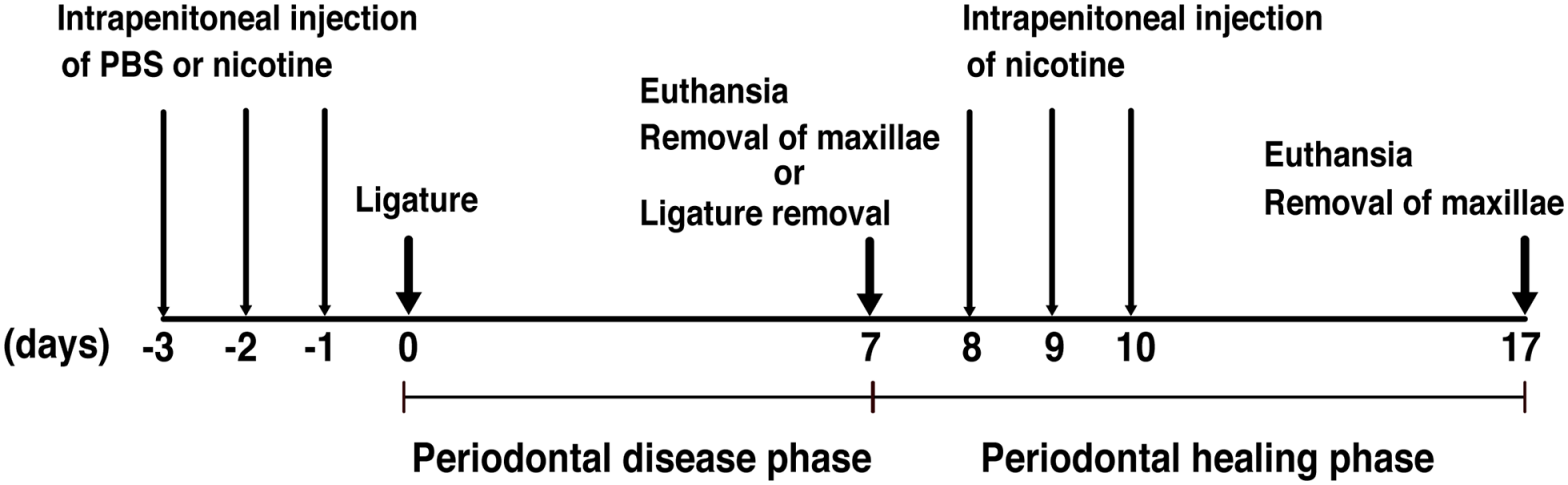
Overview of experimental design for alveolar bone repair after ligature removal (Experimental design 2). Mice were given daily intraperitoneal injections of PBS, CSC or nicotine for 3 consecutive days (days −3 to −1). Some of the mice were examined on day 7 after placement of the ligature, and some of the mice were examined 10 days after ligature removal. Three mice with nicotine-treated were given additional intraperitoneal injection of nicotine for 3 consecutive days (days 8–10) and examined 10 days after ligature removal.

**Fig 8:**
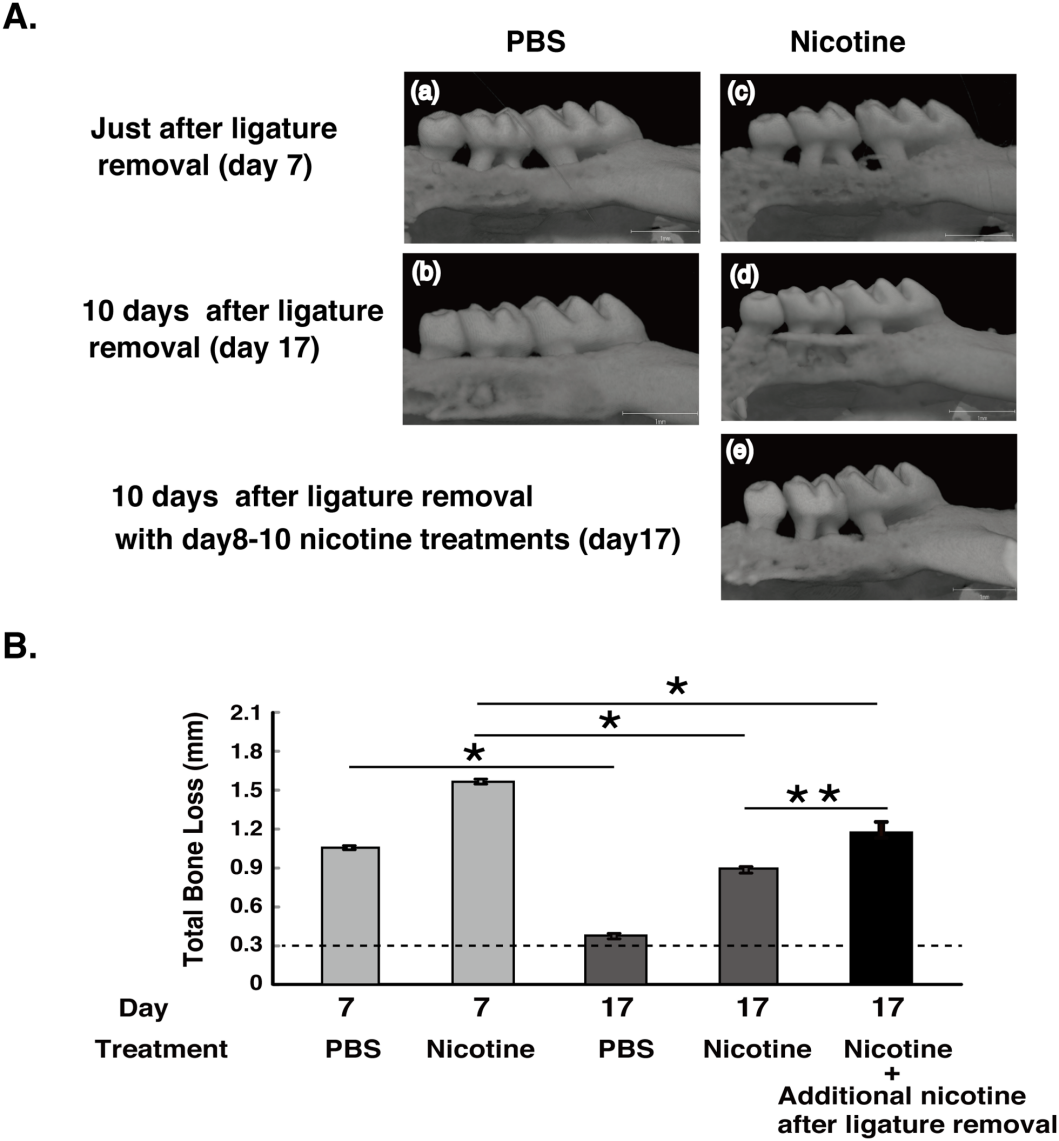
Micro-CT analysis. (A) The maxillae were scanned by micro-computed tomography. (B) Measurement of total bone loss. Bar graphs represent the mean values ± SD (n = 3). * *p* < 0.01, vs. without ligation. ** *p* < 0.01, vs. PBS-treated. Dashed line means baseline (unligated side in PBS treatment in Fig 3B).

We investigated the recovery of alveolar bone after ligature removal according to experimental design 2 (Fig 7). A large volume of mineralized bone was observed at 17 days, and the total bone loss was almost completely recovered in PBS-treated mice [Fig 8A (b) and 8B]. Although the total bone loss in the nicotine-treated mice was lower on day 17 than day 7, a large amount of total bone was still lost [Fig 8A (d)]. Additional nicotine treatment on days 8, 9, and 10 reduced the recovery of alveolar bone loss, compared with the mice without nicotine treatment after ligature removal [Fig 8A (e)]. These results suggested that nicotine not only promoted periodontal destruction but also delayed the recovery from periodontal tissue destruction.

## Discussion

This is the first study to evaluate the effects of CSC and nicotine on periodontal destruction and bone repair in ligature-induced periodontitis in mice. We have demonstrated that systemic administration of CSC and nicotine accelerated alveolar bone destruction following ligation and delayed alveolar bone recovery after ligature removal. However, our experimental approach may not reflect the inhalation of cigarette smoke by humans, which can affect local organs such as the oral and nasal cavities and lungs, which are not affected by the intraperitoneal injection of CSC and nicotine. As an environmental risk factor in periodontitis, both local and systemic effects of cigarette smoke should be intrinsically considered. Inhaled cigarette smoke is absorbed from the capillary vessels via the pulmonary alveolar epithelium and enters systemic circulation, whereas direct exposure of inhaled cigarette smoke to periodontal tissues causes vasoconstriction of the periodontal microvasculature and gingival fibrosis, which are often observed in smokers. Therefore, our study shows the systemic effects of CSC and nicotine on the progression of periodontal diseases and periodontal bone repair. Additionally, we used the ligation model to investigate the effects of smoking on periodontal destruction. The ligature model has the advantage of being able to induce alveolar bone loss within a week [18]. Although Brecx et al. reported that in large animal models, such as non-human primates, ligatures do not involve mechanical stress [21], the possibility of mechanical trauma by ligature treatment in small animals cannot be excluded because mechanical trauma could induce bone loss. Interestingly, Abe et al. reported that antibiotics treatment significantly inhibited bone loss in ligatured mice [18]. Therefore, we believe that, in this study, bacteria attached around the ligature play a major role in the induction of periodontal destruction by ligatures.

Although cigarette smoking is an important environmental risk factor in the development of periodontal diseases [12], the accumulation of a bacterial biofilm also contributes. Bosco et al. [22] reported that systemic administration of nicotine in rats caused significantly greater bone loss even in the absence of bacterial accumulation or ligation. Another study reported that nicotine enhanced the rate of bone loss in rats with ligature-induced periodontitis but had no effect on control rats [23], which is in agreement with our findings. Breivik et al [24] suggested that nicotine enhances susceptibility to periodontal destruction by inhibiting immune responses through nicotinic acetylcholine receptors. The present study evaluated ligature-induced alveolar bone loss and repair by measuring the distance between the CEJ and ABC. Cigarette smoking is associated with reduced bone mineral density (BMD) [25]; therefore, the BMD of alveolar bone may be a useful parameter for evaluating the effect of cigarette smoking in periodontal diseases.

Plaque accumulation and disease progression are exacerbated in smokers, but smokers have fewer clinical signs and symptoms of gingival inflammation, despite severe periodontal destruction, deeper probing depth, and greater attachment loss [16]. In the present study, histological analysis revealed similar levels of inflammatory cell infiltration into ligated gingival tissues after PBS, CSC, and nicotine treatment. Therefore, our findings from a mouse model are reminiscent of the clinical characteristics observed in smokers. We did not observe a quantitative difference of inflammatory cell infiltration into gingival tissues of CSC- and nicotine-treated mice. However, we assume that inflammatory cell infiltration into periodontal tissues differs qualitatively after PBS, CSC and nicotine treatment.

RANKL-expressing lymphocytes play a crucial role in alveolar bone loss in animal models [26, 27]. RANKL expression in the regional lymph nodes and spleen of mice with bacteria-induced periodontitis markedly increased compared with uninfected mice [28, 29]. In this study, RANKL expression was increased in the lymph nodes of ligated tissue in CSC or nicotine-treated mice. These results were similar to the findings regarding alveolar bone loss and distribution of osteoclasts. We did not examine the expression of osteoprotegerin, the decoy receptor of RANKL, but Buduneli et al [30] have reported that smokers with chronic periodontitis exhibit higher RANKL and lower osteoprotegerin expression in the saliva compared with nonsmoker chronic periodontitis patients. Therefore, expression of osteoprotegerin might be suppressed in CSC and nicotine-treated mice. Although we found that ligation alone did not significantly induce *Rankl* mRNA expression in our models, Takahashi et al [31] observed an increased ratio of RANKL/osteoprotegerin after the elicitation of ligature-induced experimental periodontitis. Hence, we presume that CSC and nicotine-treated mice had higher expression levels of RANKL and lower or comparable levels of osteoprotegerin in ligated periodontal tissues than PBS-treated mice. A combination of bacterial infection, such as a microbial biofilm around the ligated suture, and the systematic administration of CSC or nicotine synergistically may enhance RANKL expression in our periodontitis models. Previous *in vitro* studies reported that nicotine upregulated RANKL in human periodontal ligament (PDL) cells [32], and RANKL expression elevated by nicotine was further upregulated in human PDL cells co-cultured with CD4+ cells [33]. PBS treatment did not significantly alter RANKL expression after ligation, but significant bone loss was observed in this study. A recent study showed that a combination of tumor necrosis factor-α and interleukin-6 could differentiate osteoclast-like cells [34]. This suggests RANKL plays crucial roles in osteoclastogenesis; however, inflammatory cytokines may induce osteoclastogenesis via RANKL-independent pathways.

Although cigarette smoke contains many chemicals in addition to nicotine, our data showed that CSC and nicotine induced comparable levels of alveolar bone loss. Based on this observation, we assumed that nicotine is principally responsible for periodontal destruction in ligature-induced periodontitis and examined alveolar bone recovery after ligature removal in nicotine-treated mice. Regarding the similar amounts of bone loss recovery in PBS- and nicotine-treated mice after ligature removal, we did not examine the mechanism for these phenomena. Our results show that nicotine treatment alone before the ligature may not affect the delay of bone recovery. In fact, bone recovery was also delayed in mice that received an additional nicotine treatment after ligature removal. We assume that the metabolism of nicotine in mice may be high and that the administration of nicotine before ligature is unlikely to further affect the bone metabolism after day 7.

Our results showed that alveolar bone repair after ligature removal in nicotine-treated mice represents a valid approach and agrees with epidemiological findings in the present study that cigarette smoking impairs periodontal healing. Therefore, our experimental model may be useful for analyzing the mechanisms underlying smoking-associated periodontal diseases induced by ligation and alveolar bone healing.

In the present study, the sample size was determined based on the resource equation method [35, 36]. The properties and phenotypes of laboratory animals were homogenous, and clear differences in the amounts of bone loss among PBS-, CSC-, and nicotine-treated groups were observed in our preliminary experiments for defining the optimal experimental conditions. However, we understand that our sample sizes were small and higher numbers would improve the statistical significance of our findings.

In conclusion, we have shown that nicotine and CSC promote periodontal destruction in mice with a ligature, accompanied by increased osteoclastogenesis and RANKL expression. Furthermore, bone repair was delayed after ligature removal in nicotine-treated mice.
